# Sequential Coherence in Sentence Pairs Enhances Imagery during Comprehension: An Individual Differences Study

**DOI:** 10.1371/journal.pone.0138269

**Published:** 2015-09-18

**Authors:** Carol Madden-Lombardi, Anne-Lise Jouen, Peter Ford Dominey, Jocelyne Ventre-Dominey

**Affiliations:** 1 INSERM U846, Stem Cell and Brain Research Institute, Integrative Neuroscience Department, 18 Avenue Doyen Lépine, Bron, France; 2 CNRS (National Center for Scientific Research), Villeurbanne, France; 3 Université de Lyon, Université Lyon 1, Bron, France; Emory University, UNITED STATES

## Abstract

The present study investigates how sequential coherence in sentence pairs (events in sequence vs. unrelated events) affects the perceived ability to form a mental image of the sentences for both auditory and visual presentations. In addition, we investigated how the ease of event imagery affected online comprehension (word reading times) in the case of sequentially coherent and incoherent sentence pairs. Two groups of comprehenders were identified based on their self-reported ability to form vivid mental images of described events. Imageability ratings were higher and faster for pairs of sentences that described events in coherent sequences rather than non-sequential events, especially for high imagers. Furthermore, reading times on individual words suggested different comprehension patterns with respect to sequence coherence for the two groups of imagers, with high imagers activating richer mental images earlier than low imagers. The present results offer a novel link between research on imagery and discourse coherence, with specific contributions to our understanding of comprehension patterns for high and low imagers.

## Introduction

### Coherence in sentence processing

Our daily lives are made up of events that occur in sequences. These events are preceded or followed by other events in predictable ways, such that we comprehend situations by finding the coherence between events. Most models of language comprehension agree that the goal of comprehension is to build coherent mental representations of described events. According to the Structure Building Framework [1], this mental representation or *structure* is built through several component sub processes. First, as memory traces are activated by incoming language stimuli, these activated traces serve as the foundations for mental structures. Next, comprehenders augment their existing structures by mapping on new information when that incoming information coheres or relates to previously activated information, in which case some of the same memory traces are re-activated. When the incoming information is unrelated to previous information, mostly new memory traces are activated and comprehenders shift to initiate a new substructure. The more coherent the incoming information is with respect to previous information, the more likely the same or connected memory traces are to be activated. In contrast, the less the incoming information coheres with previous structures, the less likely the same or connected memory traces will be activated. In this case, the incoming information forms the foundation for a new substructure with a different set of active traces.

One way to measure coherence between clauses or sentences in discourse is offered by the event indexing model of Zwaan and colleagues [2–4]. According to the Event Indexing Model, events from subsequent clauses and sentences in a text are associated and integrated into long term memory based on their overlap on five situational dimensions: time, space, causation, motivation, and protagonist. The more dimensions that overlap, the stronger the connection between the two events. Likewise, a lack of overlap on multiple dimensions can lead to weak connections between events and difficulties in integration during comprehension.

Another way that events cohere is through causal patterns and repetition of frequently experienced events. Events that we experience in the real world typically follow clear patterns, and our understanding of these patterns is stored in what researchers have termed “scripts”[5,6]. A script is a structured representation of a stereotyped sequence of events in a particular context and with a particular goal. The most common example is that of the restaurant script, in which experiential knowledge about the typical sequence of events that occur when we go to a restaurant is compiled (being greeted, being seated, receiving a menu, choosing a meal, ordering the meal, waiting, receiving the meal at the table, etc.,). This script then guides understanding and interactions in the context of going to a restaurant, as well as comprehension when reading about going to a restaurant. Violations of this script (mis-orderings, unrelated events) can cause comprehension difficulties [7]. Whether we speak in terms of script violations or lack of situational overlap on multiple dimensions, when one sentence describes a protagonist performing a completely different event than in the previous sentence, there is a lack of coherence with consequences on the comprehension process. In the present study, we are interested in the effect of this (lack of) coherence on the imageability of mental representations during comprehension, so we will now turn the discussion towards imagery and the nature of mental representations.

### Perceptual representations and imagery in sentence processing

A growing body of research supports the idea that comprehenders understand event descriptions by activating simulations that incorporate perceptual information[8,9]. For instance, comprehenders are faster to verify pictures when they match the perceptual constraints of the preceding sentence, such as shape [10,11] and orientation of objects [12]. According to the simulation view of language comprehension, the words in a phrase or sentence partially reactivate traces of our experience, first activating word-level simulations that combine to yield event-specific simulations of the larger phrase or sentence. These simulations are thought to be automatic, and they may not always be available for conscious assessment, especially the lower-level (word-level) simulations. However, many comprehenders indeed report seeing a sequence of visual images expressing the semantic content of the events described through spoken or written language. These images have been shown to influence later memory [13,14], and memory for text has been shown to increase as subjects produce more images while reading [15]. Furthermore, comprehenders can be classified as high and low imagers, with high imagers showing better recognition for text than low imagers, and low imagers’ memory performance increasing when they are instructed to form visual images during reading [16–21]. This ability to form a vivid image of a described event has recently been linked to general semantic processing, as Jouen and colleagues observed increased relative pathway density issued from the temporo-parietal cortex hub of their semantic representation network for subjects who were better able to form vivid images of described events [22].

This semantic representation system appears to be cross modal, such that the meaning of sentences presented auditorily over headphones and sentences presented visually on the computer screen activate the same patterns of mental imagery. Of course, there are modality-specific processes, such as the processing of visual word forms and auditory word decoding, which activate distinct cortical areas [23]. However, higher-level semantic processes are associated with the same network of brain activation whether the language input is visual or auditory [24–29]. Studies have even shown a common semantic network for event comprehension through language and image processing [22,30–32], lending further support to behavioral results showing cross-modal priming between words and pictures [33].

### The present study

The present study investigates how the coherence between two sentences can affect the ease with which simulations are evoked when we process auditory as well as visually presented sentences. In addition, we investigate how the ease of mental imagery affects the online comprehension process in the case of reading sequentially coherent and incoherent sentence pairs. On a given trial, two sentences are presented, either over headphones or on the computer screen, and each sentence describes an event. The protagonists always overlap between two sentences, but in half of the trials, these protagonists are described as performing two events in a common sequence (i.e. within a script), and on the other half of the trials, the protagonists are described as performing two completely different events (sequential incoherence). We ask participants to rate the imageability of each sentence. Recent research has shown that subjects generally have a good metacognitive understanding of their own mental imagery and they can reliably evaluate the vividness of representations [34]. Our ratings are then used to identify two groups of comprehenders based on their self-reported ability to form vivid mental images of described events. We expect that the sequential coherence between sentences pairs will affect imageability ratings for the second sentence of a pair for both auditory and visual presentations, such that the second sentence of coherent pairs will evoke mental images more easily and quickly than that of incoherent pairs. In addition, in order to assess online comprehension, reading times will be measured for each word in the visually presented sentences. We expect that high and low imagers will show differences in online comprehension when the second sentence of a pair is sequentially incoherent vs. coherent, with high imagers showing a greater effect of coherence on reading times than low imagers. In addition, with this online methodology, we are able to test the idea that the effects of coherence may be localized to the verb of the sentence [e.g., 35].

## Method

### Participants

Thirty-one healthy, native French-speakers (16 men; mean age: 22.12; SD age: 5.65) participated in the experiment. All participants had normal or corrected-to-normal vision and no history of neurological or psychiatric disorders. The experiment was conducted in agreement with the world medical association declaration of Helsinki. The study was performed under approval (Authorization No. 10028) from the Rhône-Alpes Préfecture review board authorizing biomedical research at the Stem Cell and Brain Research Institute. Volunteers were informed about the experimental procedures before they provided written consent to participate.

### Materials

One hundred experimental sentence pairs were created, each describing agents performing everyday activities that follow scripted/predictable sequences of events (See S1 File for sample stimuli). Fifty of the sentence pairs were presented visually in the center of the computer screen, one word at a time, and fifty were presented auditorily over headphones. For both sets of fifty visually and auditorily presented sentence pairs, half (25) of the pairs described a coherent sequence of two events (e.g., “The woman waited in line at the bakery. She bought a baguette.”), and half (25) described two events that did not belong to the same coherent sequence (e.g., “The man read the menu at the restaurant. He repaired the car.”). The presentation of visual vs. auditory sentence pairs was blocked, whereas sequentially coherent and incoherent pairs were randomly presented within these blocks. Each of the 100 sentence pairs was presented both visually and auditorily (female native-French speaker), but two lists were created such that any given sentence pair was presented visually on one list and auditorily on the other list. Participants were randomly assigned to one of the two lists, assuring that participants were exposed to all 100 sentence pairs, but never saw the same sentence presented both visually and auditorily. Using the Lexique database [36], sentences were normed in terms of frequency to assure compatibility across the coherent and incoherent conditions. Sentences were also comparable in terms of grammatical constructions and number of characters per sentence (average of 71) for the two conditions, as well as the number of sentences about female vs. male protagonists, and adults vs. children.

### Procedure

During the experimental session, half of the participants saw the visually presented block of 50 sentence pairs first, and half of the participants heard the auditory block first. Whether visual or auditory, each trial began with a green fixation cross, indicating the beginning of a new sentence pair. Participants pressed the spacebar to start the trial, upon which the fixation cross disappeared and participants would either hear or see the first sentence of the pair. If the trial was part of the visual block, sentences were presented one word at a time in black font in the middle of a white screen, and this serial visual presentation was self-paced such that participants pressed the space bar to advance from word to word (response times were recorded). This differs from normal reading in that words cannot be skipped, and the words that have already been read cannot be read again (no backtracking), making it more similar to auditory presentation. In the auditory block, whole sentences were presented as wav files.

After participants had pressed the spacebar to the last word of the visually presented sentence, or after the auditorily presented sentence had finished, participants were asked how easily they were able to represent or imagine this sentence from 1 (very difficult to represent, non-vivid image) to 5 (very easy to represent, highly vivid image), using the GHJKL keys on a keyboard, relabeled 12345. This 5-point scale was chosen to remain similar to the revised version of the Vividness of Visual Imagery Questionnaire (VVIQ-2; [37]) in which the participant considers specific scenes and situations and rates the vividness of the representation along a 5-point scale. It should be noted that vividness and imageability are related but not identical, and while Denis and colleagues used the VVIQ to measure the vividness of the representation (the detail and content of the mental image; [16,17]), the emphasis in the present study was on the ease with which a sentence evokes a mental image. After their imageability response was made, the > symbol appeared, alerting participants to prepare for the second sentence of the pair. Once participants pressed the spacebar, the second sentence played over headphones (in the auditory block) or the first word of the second sentence was presented in the middle of the screen (in the visual block). When participants had pressed the spacebar to the final word of the sentence, or once the sentence wav file had finished, the same imageability rating screen appeared and participants were instructed to rate the imageability for the second sentence using the same buttons 1 through 5. These ratings and their response times were recorded. The experiment lasted approximately 40 minutes, and was run on a laptop PC with 14" display using the E-Prime stimulus presentation software [38]. At the end of the experimental session, participants responded to a 1-page questionnaire about their reading habits and their experience during the experiment (difficulty in either modality, any special strategies for representing the sentences).

### Hypotheses

1) We expected coherence between sentence pairs to affect imageability ratings such that second sentences would elicit higher imageability ratings when they describe a sequentially coherent rather than a sequentially incoherent event with respect to the preceding sentence. 2) These ratings were also expected to be made more quickly for sequentially coherent rather than sequentially incoherent events. 3) This benefit of coherence should be most marked on high imagers, as these comprehenders should have richer and faster representations to compare. 4) Given previous findings [17], it is possible that high imagers read slower than low imagers in general, as their rich representations may come at the cost of reading speed. 5) We expect that coherence will affect reading times in visually presented sentences such that sequentially coherent second events are read more quickly than sequentially incoherent second events, especially on the verb of the sentence. 6) This effect may depend on the strength and speed of the representation, such that high imagers show a greater effect of coherence on reading times than low imagers. 7) Finally, as listening and reading have been shown to produce equivalent higher-level semantic representations, the effects of imageability were not expected to differ for auditory vs. visual sentence presentations.

## Results

### Data analysis

The dependent measures of interest were the participant’s imageability rating from 1 to 5 for each sentence, as well as the time to make this rating for visually and auditorily presented sentences. The time to read each word was also a dependent measure for visually presented sentences only. As the sentences used for the sequentially coherent and incoherent conditions could not be counterbalanced because of practical constraints of the stimuli, it was necessary to take into account the length of each word on the reading time for that word in the experiment. To accomplish this, the response time for each word was divided by the number of syllables in that word. As words in the sequentially incoherent condition were compared to words in the same sentence position in the sequentially coherent condition, sentence position did not have to be controlled for. The resulting response times above or below 3 standard deviations for each condition were excluded. Likewise, for the times to make the imageability ratings, latencies over or under 3 standard deviations from a participant’s mean for a given condition were removed prior to running the analyses. This constituted removal of about 1% of the data. In addition, 4 participants were removed before analyses because they showed no variability in imageability ratings, answering 5 out of 5 on every trial without properly contemplating the task.

A K-means clustering method (k = 2 choice validated with a Bayesian Information Criteria) was used to identify the remaining 27 subjects as high imagers (n = 14; visual condition mean = 4.59, sd = 0.25; auditory condition mean = 4.58, sd = 0.26) or low imagers (n = 13; visual condition mean = 3.50, sd = 0.28; auditory condition mean = 3.63, sd = 0.51). Statistical analyses employed repeated measures analyses of variance (rmANOVA) on the imageability ratings, the times to make these ratings, and reading times for individual words in visually presented sentences. The first two measures were subjected to a rmANOVA incorporating the within-subjects factors *modality* (visual vs. auditory presentation), *coherence* (sequential vs. non-sequential sentence pairs) and *sentence order* (first vs. second sentence), and the between-subjects factor *imageability group* (high vs low imagers). ANOVAs were initially run to include the presentation order of block modality (visual or auditory block first) but no significant effects of block order were observed, and as this factor had no theoretical contribution to the study, it was removed from the analysis (43% of participants classified as low-imagers had seen the visual block first, and 69% of high imagers had seen the visual block first). Word reading times for second sentences in the visual condition were submitted to a rmANOVA including the within-subjects factors *coherence* (sequential vs. non-sequential sentence pairs) and *word order* (word1, word2, word3, word4), as well as the between-subjects factor *imageability group* (high vs low imagers). Specific effects for each factor were identified by Bonferroni post-hoc analysis, and significance level was established at a 95% confidence interval.

### Imageability Ratings

The first dependent variable of interest is the imageability rating, for which means and standard deviations are displayed in Table 1. While most participants maintained relatively high imageability ratings (all stimuli were pre-tested to be highly imageable), we were able to distinguish two patterns of responses, corresponding to high and low imagers (Table 1). The difference between these two groups was of course highly significant [F(1,25) = 79.0, p < 0.001] regardless of visual or auditory presentation.

**Table 1:** Means for imageability ratings by group, modality, coherence, and sentence order.

	VISUAL				AUDITORY			
	Sequential		Non-sequential		Sequential		Non-sequential	
	Sent 1	Sent 2	Sent 1	Sent 2	Sent 1	Sent 2	Sent 1	Sent 2
**high imagers n = 14**	4.71	4.77	4.62	4.17	4.66	4.83	4.68	4.04
**low imagers n = 13**	3.49	3.89	3.54	3.08	3.70	4.04	3.59	3.19
**Total n = 27**	4.08	4.31	4.06	3.60	4.16	4.42	4.12	3.60

**Fig 1:**
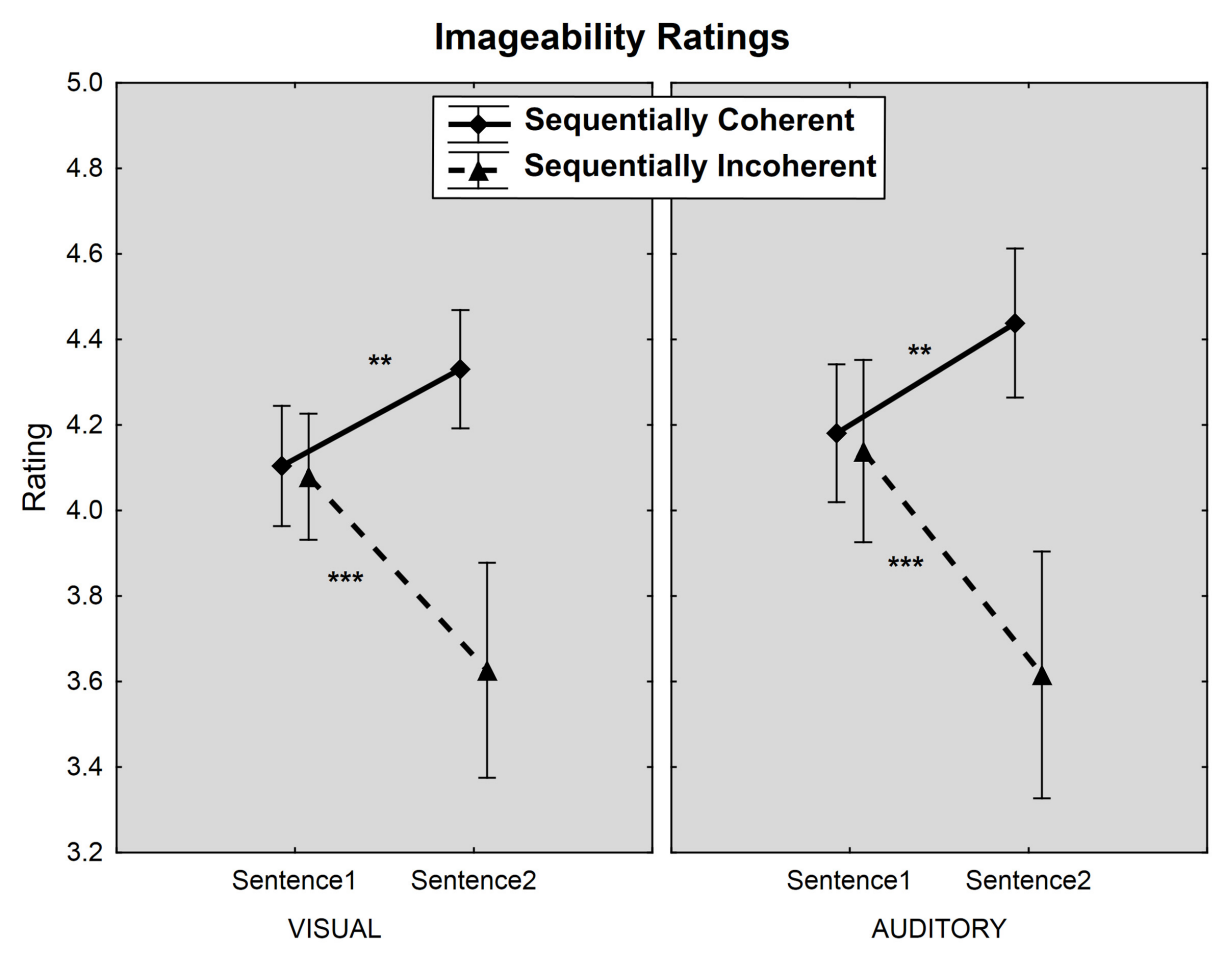
Mean imageability ratings for sequentially coherent and incoherent sentence pairs in the visual and auditory conditions. Bars: Standard deviations (*** p< 0.001, ** p< 0.01).

The rmANOVA showed a clear main effect of coherence on the imageability ratings [F(1,25) = 27.4, p < 0.001], such that the sentences from sequentially coherent pairs were rated as more imageable than sentences from sequentially incoherent pairs. This effect was qualified by a significant interaction between coherence and sentence order (Fig 1) [F(1, 25) = 22.4, p < 0.001], wherein the effect of coherence, as expected, was only present on the second sentence of the pair (Bonferroni: p < 0.001), and not on the first sentence (Bonferroni: p = 1).

These effects were further qualified by a four-way interaction among coherence, sentence order, modality, and group [F(1,25) = 4.4; p < 0.05]. This interaction was largely driven by a ceiling effect for high imagers, whereby their already high imageability ratings did not increase from the first to the second sentence in sequentially coherent pairs in both presentation modalities, while ratings for the low imagers increased significantly from first to second sentences of coherent pairs both in the visual (Bonferroni: p < 0.001) and auditory (Bonferroni: p < 0.005) modalities. In contrast, for sequentially incoherent pairs, both high and low imagers’ ratings significantly decreased from first to second sentences in both visual (Bonferroni: p < .001) and auditory (Bonferroni: p < 0.001) modalities. Furthermore, second sentences yielded significantly higher imageability ratings for sequentially coherent than for incoherent sentences in both modalities for both groups (all Bonferroni: p < 0.001).

### Response times for imageability ratings

**Fig 2:**
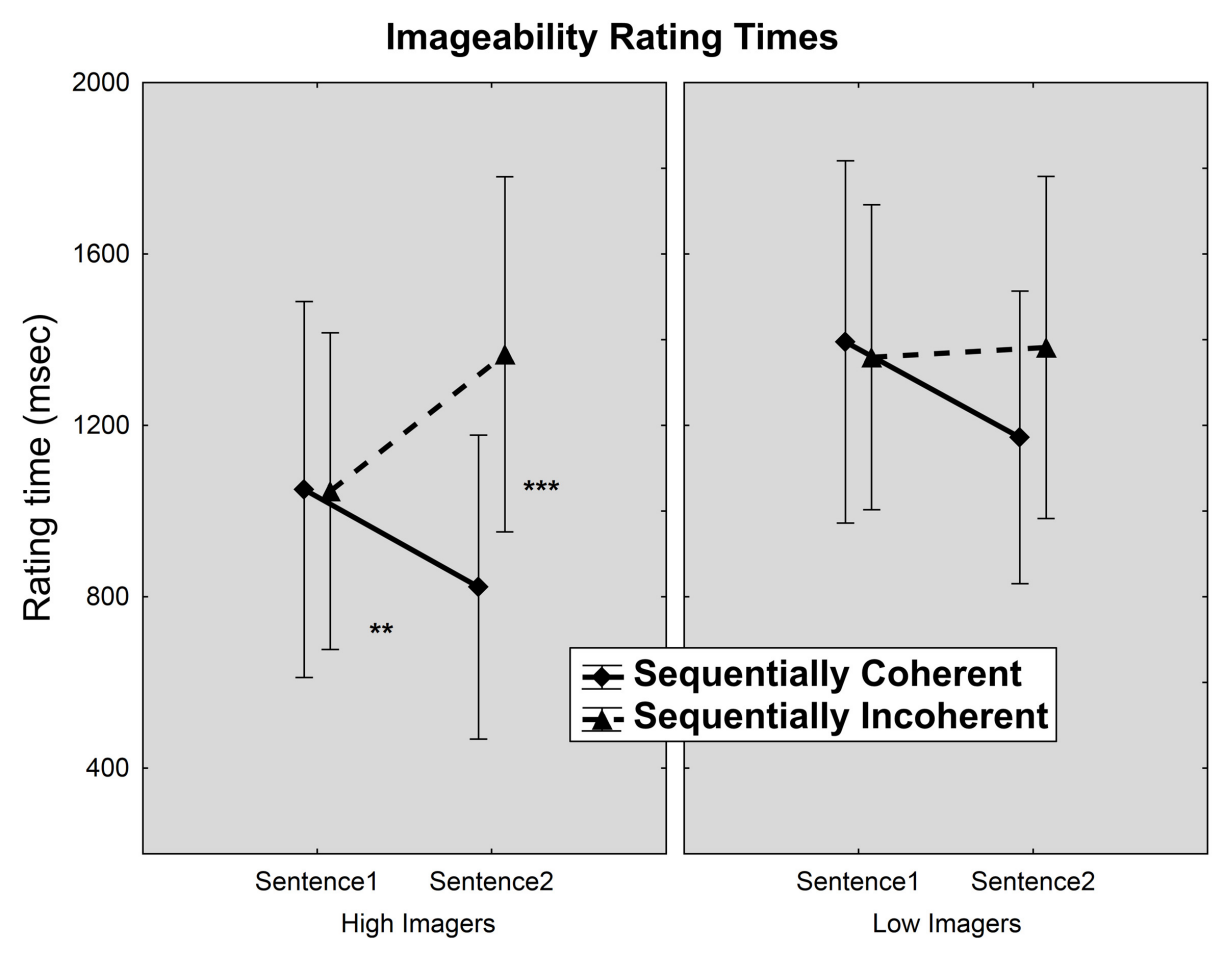
Mean times in milliseconds for high and low imagers to provide imageability ratings for sequentially coherent and incoherent sentence pairs. Bars: Standard deviations (*** p < 0.001, ** p < 0.01).

Response times to make imeageability ratings are presented in Fig 2. As observed with the imageability ratings, the times to make these ratings also showed a significant main effect of coherence [F(1,25) = 21.9, p < 0.001], which was qualified by an interaction between coherence and sentence order [F(1, 25) = 31.6, p < 0.001]. While there were no coherence differences on rating times on the first sentence, rating times were faster for sequentially coherent rather than sequentially incoherent second sentences in both modalities (Bonferroni: p < 0.001). The coherence effect was also qualified by an interaction between coherence and group [F(1,25) = 5.7, p < 0.05] and a three-way interaction among coherence, group and order [F(1,25) = 4.6, p < 0.05]. Only for high imagers and only on the second sentence, rating times were faster for sequentially coherent sentences compared to incoherent sentences (Bonferroni: p < 0.001). Likewise, only ratings from high imagers on coherent pairs yielded a difference between first and second sentences (Bonferroni: p < 0.01).

### Word reading times for visually presented sentences

**Fig 3:**
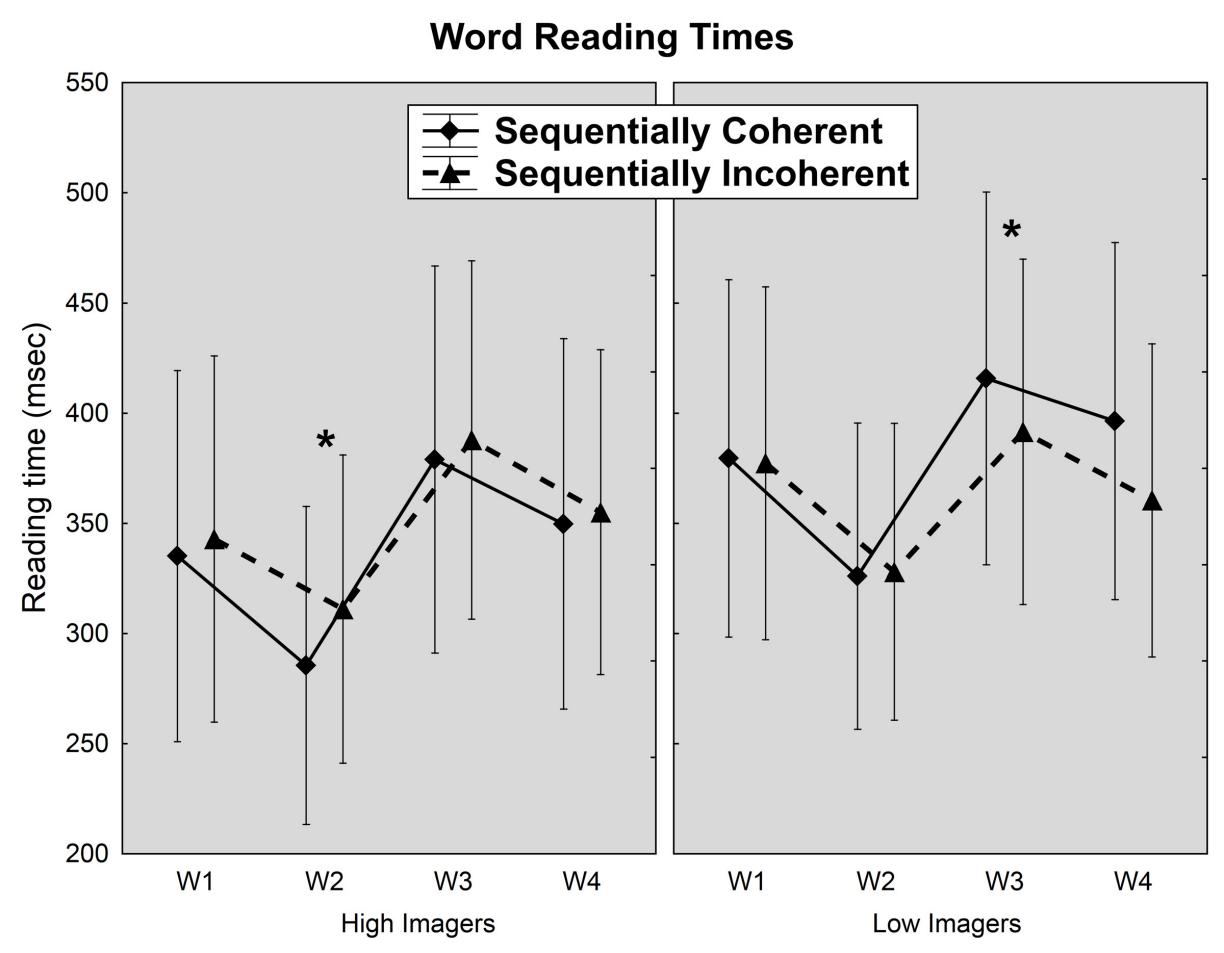
Reading times in milliseconds for individual words (divided by number of syllables per word) for high and low imagers in sequentially coherent and incoherent sentences. Bars: Standard deviations (* p< 0.05).

The rmANOVA for word reading times on the second sentences yielded an interaction between coherence and word order [F(3,75) = 5.2, p < 0.01] as well as an interaction between coherence and group [F(1,25) = 6.3, p < 0.05]. Upon examination of each word of the second sentence separately, only the second and third words showed effects of coherence. The second word (generally the verb, on over 80% of trials) showed an effect of coherence [F(1,25) = 5.5, p = 0.027], which was qualified by a marginally significant interaction between coherence and group [F(1,25) = 4.02, p = 0.056], whereby only the high imagers showed a speedup in reading times on this second word for sequentially coherent compared to sequentially incoherent pairs (Bonferroni: p < 0.05). Inversely, the third word also showed an interaction between coherence and group [F(1,25) = 10.0, p < 0.01] whereby only low imagers showed faster reading times on this third word for sequentially incoherent compared to sequentially coherent pairs (Bonferroni: p < 0.05), as illustrated in Fig 3.

## Discussion

In the present study participants read or heard pairs of sentences describing two sequentially coherent events or two sequentially incoherent events and they provided imageability ratings. Results showed clear effects of sequence coherence on participants’ ability to create a vivid image of described events. In general, imageability ratings were higher and faster when a pair of events was sequentially coherent rather than when the pair described two sequentially incoherent events, especially for high imagers, despite a ceiling effect for sequentially coherent ratings. In addition, high imagers showed slower reading times on the verb of sequentially incoherent sentences relative to coherent sentences, whereas low imagers showed faster reading times on sequentially incoherent relative to coherent words following the verb, suggesting different comprehension processes for high and low imagers. The modality of sentence presentation did not yield major differences, suggesting that higher-level semantic representations are cross modal, at least in the context of this visual imagery evaluation.

The fact that imageability ratings were higher and faster for sequentially coherent rather than incoherent sentence pairs suggests that comprehenders are more quickly able to construct a richer representation when an event description is coherent with previously comprehended information. This effect is generally consistent with previous research [39] and fits with theories of language comprehension that postulate facilitation for incoming information that overlaps with previous linguistic input [1–3,7]. In interpreting the results it is important to keep in mind that subjects were asked to rate their ease of imageability for each sentence, which may indeed have artificially inflated the use of imagery during the comprehension task. While this may shift the task a bit further along the continuum from automatic activated simulations in the direction of deliberate imagery, the relative differences (coherent vs. incoherent, high vs. low imagers) are clearly informative. Furthermore, the differences in online reading times to individual words also suggest that the imagery invoked in the present study is more in line with rapid real-time sentence processing.

It is also important to correctly characterize the difference in coherence between the sentences in sequentially coherent and sequentially incoherent pairs. The protagonists are always overlapping, but in sequentially coherent pairs, they are described as performing two events in the same sequence, and on sequentially incoherent pairs, the protagonists are described as performing two completely different events. In terms of the Event-Indexing Model [2–4], all dimensions overlap in the case of sequentially coherent sentence pairs, whereas most or all dimensions except for the protagonist change in the case of sequentially incoherent sentence pairs. Because the protagonists are always re-referenced, this second case constitutes more of a shift in time or storyline rather than a completely incoherent or unrelated event. In the context of a list of events that a protagonist did throughout the day, two non-sequential events would not be considered incoherent. However, as the two sentences are presented in isolation, the comprehender most likely expects a continuation of the initially described event, so a second sentence that shifts to a new event may be interpreted as something of a script violation, especially in cases where the first event is part of a stereotyped situation. Even when the initial event is not part of a scripted situation, the sequentially incoherent second sentence will lack sufficient connections to the preceding sentence to be integrated and a new representational structure must be initiated.

Regardless whether we speak in terms of script violations, initiating a new substructure, or lack of overlap on multiple dimensions of a situation model, it is clear that sequentially incoherent second sentences were not well integrated with the previous sentences, giving rise to slower and lower imageability ratings. There are several possible mechanisms that may underlie this slowdown and decrease in imageability ratings. A cognitive search effort to resolve the coherence violation could cause interference with (or recruit resources away from) the activation of a simulation of the event. Alternatively, the facilitated imageability in coherent sentences could result from a combined representation (and thus a richer image) of both sentences of the coherent pair as compared to a less rich representation of only the second sentence in sequentially incoherent pairs. However, while this combined representation may be richer, it does not necessarily yield shorter rating times. It is also possible that the imageability ratings might be affected by lexical priming between the two sentences, which may have been greater in sequentially coherent pairs, but this would not explain the differences between high and low imagers. The most probable explanation is that the second sentence of a coherent pair may be, to a certain extent, already included in the representation evoked by the first sentence. For instance, if the first sentence describes a woman in line at the bakery, the reader/listener (especially high imagers) may activate a simulation of this situation that includes her buying bread. Then, when the second sentence is presented (“She bought a baguette”), the process of creating a mental image should be easy and rapid, as it was mostly completed already.

While the underlying mechanism of the imagery facilitation remains speculative, recent findings suggest that lexical priming and mental imagery effort may be able to be disentangled. Brouwer and colleagues propose that the N400 is linked to lexical retrieval rather than semantic integration, and therefore is an index of lexical priming [40]. Alternatively, the P600 component is argued to reflect the effort in reworking an initial mental representation in terms of discourse semantics, rather than the revision of a syntactic analysis as traditionally viewed [40]. Even in the absence of syntactic difficulty, a larger P600 is observed when a novel discourse referent has to be established [41], or an instrument has to be inferred [41,42]. This could be an index of the effort required to activate a discourse-appropriate mental image of the incoming sentence, regardless of lexical priming. When some aspects of the described situation are already provided by the previous sentence, this effort will be reduced, producing smaller P600s as well as shorter reading times as seen here. EEG experiments on the current stimuli set are underway to further investigate the mechanisms underlying the observed imagery facilitation.

Aside from their ceiling effect on imageability ratings, the observed effects of coherence were stronger for high imagers than low imagers, especially in rating times. This suggests that high imagers are more quickly able to activate rich simulations of the described events than low imagers, and therefore they are more sensitive to effects of sequence coherence as they compare their rich representations. No other measures of comprehension ability (reading span, working memory, vocabulary measures) were collected in the current study, so it is not possible to determine whether imageability capabilities might be linked to general comprehension skill. However, sensitivity to coherence in imageability of the stimuli is suggestive of general language ability, and it could be that the ability to activate a rich, vivid simulation is closely associated with or even partly responsible for comprehension skill in general. Indeed, using this same imageability measure in a combined fMRI and DTI study, Jouen and colleagues [22] observed a correlation between the relative pathway density originating from the temporo-parietal cortex for a given participant and that participant’s mean imageability ratings for sentences similar to those used in the present study. This suggests a potential functional link between the ability to activate vivid simulations of described events and the strength of temporo-parietal connections in a brain network for semantic representation.

High imagers showed a speedup in reading times for sequentially coherent compared to sequentially incoherent pairs on the second word of the sentence, which was the verb on over 80% of trials. This suggests that the high imagers were more sensitive to the coherence between the two sentences earlier than low imagers, perhaps because their event simulations were more vivid or were activated more quickly. This is consistent with the idea that high imagers are more likely to activate a simulation of the first sentence that also includes the situation described in the second sentence. Thus, when a coherent second sentence is encountered, the simulation is already largely in place and only needs to be verified or slightly modified, whereas the simulation for an incoherent second sentence would have to be constructed entirely. It is important that this coherence sensitivity is localized to the verb of the sentence, as verbs have been shown to drive event simulations [35], and this was the main source of disambiguating information about the event described in the second sentence.

While the low imagers do not show this sensitivity on the second word of the sentence, they do show sensitivity to the incoherence on the following word, manifest in faster reading times for sequentially incoherent compared to sequentially coherent pairs on the third word of the sentence. This somewhat counter-intuitive finding can also be explained by the idea explained above that low imagers are less likely to activate a simulation that includes both the action described in the first sentence (waiting in line at the bakery), as well as the probable subsequent action (buying bread). In this case, a coherent second sentence still requires the activation of a simulation of buying bread, as well as the integration of the two coherent simulations, which causes higher imageability ratings but also a slowdown just after the verb of the sentence. In the case of an incoherent second sentence, the simulation is also activated, but the integration is not required, probably due to an abandonment of the search-after-meaning process [43]. Under certain circumstances, readers may abandon the effort to establish or maintain coherence. For instance, when they consider the input to be completely lacking in global coherence, no effort is made to resolve that lack of coherence. They might also abandon the effort to establish coherence when they do not possess the resources or relevant knowledge, or if their goals do not require them to do so. In the present study, low imagers may have lacked the resources (vividness or speed of simulation) to establish coherence between the two sequentially incoherent sentences, so that they abandoned the effort to do so, and increased their reading speed immediately following the critical point of the sentence, the verb. Alternatively, low imagers may have had lower self-imposed goals for constructing a coherent representation of the sentence pair, or they may have found the sequentially incoherent pairs less related than high imagers did, so that they were more likely than high imagers to abandon efforts to maintain coherence. Although our data do not allow us to determine the underlying cause, it is probable that the low imagers abandoned the search-after-meaning process just after the point of disambiguation (the verb) on sequentially incoherent sentences, and this caused the speedup just after the verb relative to sequentially coherent sentence pairs. The flip side of this is that low imagers spent more time and effort integrating sentence pairs when they were sequentially coherent, which is consistent with Denis’ finding [17] that rich imagery comes at a cost to reading speed. Only when the second sentence is judged to be incoherent do low imagers seem to forego this mental effort.

The fact that low imagers show a later effect of coherence than high imagers may suggest that imageability is linked to other general measures of comprehension. For instance, the current finding is consistent with previous research in which low span comprehenders were slower to activate perceptual representations than high span comprehenders [10]. In that study, high and low span comprehenders responded to pictures that either matched or mismatched a target object’s shape (flying or perched eagle) as implied by the preceding sentence context (“In the sky/nest there was an eagle.”). When pictures were presented 750 ms after the target object was mentioned as the last word of the sentence, both high and low span comprehenders showed faster responses for the matching picture, demonstrating that they had activated a contextually appropriate perceptual representation of the target object. However, immediately upon hearing the target object mentioned as the last word of the sentence, only high span comprehenders demonstrated the match effect, whereas low span comprehenders required more processing time before the perceptual representation was activated. Interestingly, and similar to the current study, low span comprehenders took longer to respond at the later interval when their perceptual representation had been activated than at the immediate interval when they had not yet activated a perceptual representation.

On a more general note, the fact that sequential coherence between sentences can impact the imageability of representations has implications for experimental psychology methodologies, especially with respect to perceptual simulations in language comprehension. If simulations during language comprehension become easier and more vivid as the sequential relation between events increases, then experiments that present unrelated sentences (or even words) in isolation may evoke less rich simulations. This study suggests that lists of unrelated stimuli (words, sentences) should yield weaker perceptual effects [11] and perhaps motor effects [44] than larger texts and naturalistic “in context” language stimuli. Likewise, within any subject population, perceptual tests of mental simulations may produce weaker effects for those participants who are low imagers. These factors should be taken into account when testing for perceptual (and perhaps motor) differences in mental simulations.

In conclusion, the present study shows that sequential coherence between two sentences in a pair affects the perceived ability to form mental images of the sentence pairs. Imageability ratings were higher and faster when a pair of events was sequentially coherent rather than when the pair described two sequentially incoherent events, especially for high imagers. This effect held for both auditory and visual presentations. These ratings, in addition to reading times on individual words, suggested different levels of simulation for high and low imagers, with high imagers more likely to simulate both events in a sequence when the first is presented. Low imagers seem to limit their simulation to the currently described event, and seem more likely to abandon efforts to establish links between the sequentially incoherent sentences. While further research is required to better understand the specific mechanisms underlying the relationship between coherence in event sequences and imageability, it is clear that sequential coherence can impact the imageability of described events, and the ability to vividly simulate events can impact comprehension patterns for coherent and incoherent stimuli.

## Supporting Information

S1 File Sample Stimuli. S1 File provides examples of sentence pairs in the sequentially coherent and sequentially incoherent conditions.
(DOCX)

S2 File Full data set. S2 File displays the full set of individual subject means for all measures used in the current study.
(XLSX)

## References

[1] Gernsbacher M.A. (1997). Two decades of structure building, Discourse Processes 23: 265–304. 25484476 10.1080/01638539709544994 PMC4255941

[2] Zwaan R.A., Langston M.C., Graesser A.C. (1995). The construction of situation models in narrative comprehension: An event-indexing model. Psychological Science 6: 292–297.

[3] Zwaan R.A., & Radvansky G.A. (1998). Situation models in language comprehension and memory. Psychological Bulletin 123: 162–185. 9522683 10.1037/0033-2909.123.2.162

[4] Zwaan R.A. (1999). Five dimensions of situation-model construction In: Narrative comprehension, causality, and coherence: Essays in honor of Tom Trabasso, Goldman S.R., Graesser A.C., van den Broek P. (eds). Erlbaum: Mahwah, NJ; 93–110.

[5] Schank R.C. & Abelson R.P. (1977). Scripts, plans, goals, and understanding Lawrence Erlbaum, Hillsdale, NJ.

[6] Read S.J. (1987). Constructing causal scenarios: A knowledge structure approach to causal reasoning. Journal of Personality and Social Psychology, 52, 288–302. 3559892 10.1037//0022-3514.52.2.288

[7] Lorch R.F. Jr, Lorch E.P., & Matthews P.D. (1985). On-line processing of the topic structure of a text. Journal of Memory and Language, 24, 350–362.

[8] Barsalou L.W. (1999). Perceptual Symbol Systems. Behavioral and Brain Sciences, 22, 577–660. 11301525 10.1017/s0140525x99002149

[9] Zwaan R.A., & Madden C.J. (2005). Embodied sentence comprehension In Pecher D., & Zwaan R.A. (eds). The grounding of cognition: The role of perception and action in memory, language, and thinking. Cambridge, UK: Cambridge University Press.

[10] Madden C.J. & Zwaan R.A. (2006). Perceptual Representation as a Mechanism of Lexical Ambiguity Resolution: An Investigation of Span and Processing Time. Journal of Experimental Psychology: Learning, Memory, and Cognition, 32, 1291–1303. 17087584 10.1037/0278-7393.32.6.1291

[11] Zwaan R.A., Stanfield R.A., Yaxley R.H. (2002). Do language comprehenders routinely represent the shapes of objects? Psychological Science, 13, 168–171. 11934002 10.1111/1467-9280.00430

[12] Stanfield R.A., & Zwaan R.A. (2001). The effect of implied orientation derived from verbal context on picture recognition. Psychological Science, 12, 153–156. 11340925 10.1111/1467-9280.00326

[13] Denis M. (1979). Les images mentales Paris: Presses Universitaires de France.

[14] Paivio A. (1971). Imagery and verbal processes New York: Holt, Rinehart, & Winston.

[15] Anderson R.C., & Kulhavy R.W. (1972). Imagery and prose learning. Journal of Educational Psychology, 63, 242–243.

[16] Chaguiboff J., & Denis M. (1981). Activité d’imagerie et reconnaissance de noms provenant d’un texte narratif. L’Année Psychologique, 111, 69–86.

[17] Denis M. (1982). Imaging while reading text: A study of individual differences Memory & Cognition, 10(6), 540–545.

[18] Kulhavy R.W., & Swenson I. (1975). Imagery instructions and the comprehension of text. British Journal of Educational Psychology, 45, 47–51.

[19] Lesgold A.M., McCormick C., & Golinkoff R.M. (1975). Imagery training and children’s prose learning. Journal of Educational Psychology, 67, 663–667.

[20] Levin J.B., & Divine-Hawkins P. (1974). Visual imagery as a prose-learning process. Journal of Reading Behavior, 6, 23–30.

[21] Pressley G.M. (1976). Mental imagery helps eight-year-olds remember what they read. Journal of Educational Psychology, 611, 355–359.

[22] Jouen A−L., Ellmore T.M., Madden C.J., Pallier C., Dominey P.F., & Ventre−Dominey J. (2015). Beyond the word and image: Characteristics of a common meaning system for language and vision revealed by functional and structural imaging. NeuroImage, 106 72–85. 10.1016/j.neuroimage.2014.11.024 25463475

[23] Cohen L., Lehéricy S., Chochon F., Lemer C., Rivard S., Dehaene S. (2002). Language-speciﬁc tuning of visual cortex? Functional properties of the visual word form area. Brain, 125, 1053–1069. 10.1093/brain/awf094 11960895

[24] Booth J.R., Burman D.D., Meyer J.R., Gitelman D.R., Parrish T.B., Mesulam M.M. (2002). Modality independence of word comprehension. Human Brain Mapping, 16, 251–61. 12112766 10.1002/hbm.10054 PMC6871904

[25] Buchweitz A., Mason R.A., Tomitch L.M.B., & Just M.A. (2009). Brain activation for reading and listening comprehension: An fMRI study of modality effects and individual differences in language comprehension. Psychology & Neuroscience, 2, 111–123. 10.3922/j.psns.2009.2.003 PMC3081613 21526132

[26] Carpentier A., Pugh K.R., Westerveld M., Studholme C., Skrinjar O., Thompson J.L., et al (2001). Functional MRI of language processing: Dependence on input modality and temporal lobe epilepsy. Epilepsia, 42, 1241–1254. 11737158 10.1046/j.1528-1157.2001.35500.x

[27] Constable R.T., Pugh K.R., Berroya E., Mencl W.E., Westerveld M., Ni W., et al (2004). Sentence complexity and input modality effects in sentence comprehension: an fMRI study. NeuroImage, 22, 11–22. 15109993 10.1016/j.neuroimage.2004.01.001

[28] Jobard G. Crivello F, Tzourio-Mazoyer N. (2003). Evaluation of the dual route theory of reading: a metanalysis of 35 neuroimaging studies. NeuroImage, 20, 693–712. 14568445 10.1016/S1053-8119(03)00343-4

[29] Michael E.B., Keller T.A., Carpenter P.A., Just M.A. (2001). fMRI investigation of sentence comprehension by eye and by ear: modality fingerprints on cognitive processes. Human Brain Mapping, 13, 239–252. 11410952 10.1002/hbm.1036 PMC6872122

[30] Kircher T., Sass K., Sachs O., & Krach S. (2009). Priming words with pictures: neural correlates of semantic associations in a cross-modal priming task using fMRI. Human Brain Mapping, 30(12), 4116–4128. 10.1002/hbm.20833 19530217 PMC6870681

[31] Van Doren L., Dupont P., De Grauwe S., Peeters R., & Vandenberghe R. (2010). The amodal system for conscious word and picture identification in the absence of a semantic task. Neuroimage, 49(4), 3295–3307. 10.1016/j.neuroimage.2009.12.005 20004724

[32] Vandenberghe R., Price C., Wise R., Josephs O., & Frackowiak R. S. (1996). Functional anatomy of a common semantic system for words and pictures. Nature, 383(6597), 254–256. 8805700 10.1038/383254a0

[33] Bajo M.T. (1988). Semantic facilitation with pictures and words. Journal of Experimental Psychology: Learning, Memory & Cognition, 14(4), 579–589. 10.1037//0278-7393.14.4.579 2972797

[34] Pearson J., Rademaker R.L., & Tong F. (2011). Evaluating the mind's eye: the metacognition of visual imagery. Psychological Science, 22, 1535–42. 10.1177/0956797611417134 22058106

[35] Zwaan R.A., & Taylor L. (2006). Seeing, acting, understanding: Motor resonance in language comprehension. Journal of Experimental Psychology: General, 135, 1–11. 16478313 10.1037/0096-3445.135.1.1

[36] New B., Pallier C., Brysbaert M., & Ferrand L. (2004). Lexique 2: A new French lexical database. Behavior Research Methods, Instruments, & Computers. 36 (3), 516–524. 10.3758/bf03195598 15641440

[37] Marks D.F. (1995). New directions for mental imagery research. Journal of Mental Imagery, 19, 153–167.

[38] Schneider W., Eschman A., & Zuccolotto A. (2002) E-Prime User’s Guide. Pittsburgh: Psychology Software Tools Inc.

[39] Bransford J.D., & Johnson M.K. (1972). Contextual prerequisites for understanding: Some investigations of comprehension and recall. Journal of Verbal Learning and Verbal Behavior, 11, 717–726.

[40] Brouwer H., Fitz H. & Hoeks J. (2012) Getting real about Semantic Illusions: Rethinking the functional role of the P600 in language comprehension. Brain Research, 1446, 127–143. 10.1016/j.brainres.2012.01.055 22361114

[41] Burkhardt P. (2007). The P600 reflects cost of new information in discourse memory. Neuroreport 18, 1851–1854. 18090325 10.1097/WNR.0b013e3282f1a999

[42] Schumacher P.B. (2011). The hepatitis called…: electrophysiological evidence for enriched composition In: Meibauer J., Steinbach M. (Eds.), Experimental Pragmatics/ Semantics. John Benjamins, Amsterdam, NL, pp. 199–219.

[43] Graesser A.C., Singer M., & Trabasso T. (1994). Constructing inferences during narrative text comprehension. Psychological Review, 101 (3), 371–395. 7938337 10.1037/0033-295x.101.3.371

[44] Glenberg A.M., & Kaschak M.P. (2002). Grounding language in action. Psychonomic Bulletin and Review, 9, 558–565. 12412897 10.3758/bf03196313

