# Supplementary material for: Sequential Coherence in Sentence Pairs Enhances Imagery during Comprehension: An Individual Differences Study
Source: PLoS One. 2015 Sep 18;10(9):e0138269. doi: 10.1371/journal.pone.0138269 (PMC4575052; doi:10.1371/journal.pone.0138269)
Supplement: S1 File — provides examples of sentence pairs in the sequentially coherent and sequentially incoherent conditions. (DOCX) [file pone.0138269.s001.docx]

S1 File. Sample Stimuli

Sequentially coherent sentence pairs

Le garçon met les écouteurs dans ses oreilles. Il allume son lecteur mp3.

The boy puts his headphones on his ears. He turns on his mp3 player.

L'homme verse du produit vaisselle dans l'évier. Il lave la vaisselle avec une éponge.

The man pours dish detergent into the sink. He washes the dishes with a sponge.

La petite fille allume la télévision. Elle regarde un dessin animé.

The little girl turns on the television. She watches a cartoon.

La jeune femme plie le linge propre. Elle le range dans le placard.

The young woman folds the clean laundry. She puts it away in the closet.

Sequentially incoherent sentence pairs

La petite fille ouvre la porte du réfrigérateur. Elle poste la lettre.

The little girl opens the refrigerator door. She mails the letter.

L'homme lit le menu dans le restaurant. Il répare la voiture.

The man reads the menu at the restaurant. He repairs the car.

La femme entre dans la pharmacie. Elle lance un dé à jouer.

The woman walks into the pharmacy. She rolls the dice.

Le jeune homme allume l'ordinateur. Il essuie la vaisselle.

The young man turns on the computer. He dries the dishes.
